# Low temperature SiC die-attach bonding technology by hillocks generation on Al sheet surface with stress self-generation and self-release

**DOI:** 10.1038/s41598-020-66069-8

**Published:** 2020-06-03

**Authors:** Chuantong Chen, Katsuaki Suganuma

**Affiliations:** 1 Laboratory of flexible and power three dimensional system integration, Osaka university, Ibaraki-shi Osaka, 567-0047 Japan; 2 The institute of scientific and industrial research, Osaka university, Ibaraki-shi Osaka, 567-0047 Japan

**Keywords:** Materials for devices, Structural materials, Techniques and instrumentation, Theory and computation

## Abstract

This paper introduced an approach of die-attach bonding technology based on a low-cost high-purity aluminum (99.99%) sheet in a silicon carbide (SiC)/direct bonded aluminum (DBA) power module. Both sides of an Al sheet were sputtered by a thin Ti and Ag layer, which generated a tensile stress of 166 MPa on the Al surface. After heating, the Al surface displayed a large quantity of Ag hillocks by stress self-release due to the coefficient of thermal expansion (CTE) mismatch among Al, Ti, and Ag. The SiC/Al sheet/DBA substrate interfaces were bridged by the generation of these hillocks, which correspond to a robust shear strength of 33.4 MPa in a low-temperature process. Hillocks generation and the interface bonding mechanism by surface stress self-generation and self-release were systematically analyzed by scanning electron microscopy (SEM), X-ray diffraction (XRD), and transmission electron microscopy (TEM). The shear strength remains constant at 32.1 MPa after high-temperature storage at 250 °C for 500 h, which suggests that the Al sheet possesses excellent high-heat resistance and thermal stability. This novel approach of die-attach bonding technology serves as an attractive alternative for SiC power devices that require high-temperature performance.

## Introduction

Silicon carbide (SiC) forms a next-generation power semiconductor that realizes innovative energy saving in all types of electronic equipment, such as information communication equipment, electric vehicles, and electric railways^1–4^. Next-generation super-heat-resistant packaging needs to secure reliability at extremely high operating temperatures (maximum temperature of 250 °C), which are substantially higher than the operating temperature of conventional Si devices (maximum temperature of 150 °C). Although the junction temperature of SiC is 250 °C, SiC can operate at high temperatures. The heat resistance of each part that constitutes the module is lower than 175 °C, which is representative of most solder die-attach materials. To address these challenges, a novel class of die-attach technologies is required due to the processability, compliance, and cost of solders. However, the electrical and thermal properties must match those of a pure metal.

SiC power device modules comprise several components, including terminals, a SiC die, direct bonded copper (DBC) or direct bonded aluminum (DBA) substrate, and a heat sink. The bonding technology of SiC die devices, which can be employed for high operating temperature mounting, can be divided into three categories: (1) high melting point solder bonding^5–7^, (2) solid–liquid inter-diffusion bonding (SLID)^8–10^, (3) sinter silver paste joining^11–15^ or copper paste joining^16,17^. SLID bonding technology has a few advantages, such as a relatively short bonding time and some surface roughness tolerance; however, it is generally limited to material combinations with a favorable phase diagram^17^ and interface voids generation^18^. The use of sintered Ag paste as a bonding layer has received considerable attention due to its superior electrical and thermal performance, high temperature stability^19^, and crack self-healing function^20^. However, complex printing processes and high costs have become major obstacles for industrial applications. In addition, Ag is expensive and easily corroded due to vulcanization in air, both of which hinder its extensive application in electric devices^21^. Sintered Cu particles are less costly than Ag particles and use the same element as the Cu circuit to which they must be joined. However, the joining condition and processes are problematic due to oxidation^17^.

Recently, a solid-state bonding technology that uses porous Ag^22–24^ or Ag foils^25,26^ has been reported for high-temperature applications. The bonding mechanism is attributed to solid interface inter-diffusion, which requires high-temperature or high-pressure conditions^24,26^, which contribute to high costs. A direct bonding method of Ag coating on the Si chip surface has been reported. This Ag-Ag coating layer can be bonded, referred to as “Ag Direct Joining” or “Stress Migration Joining”^27–29^, achieving a satisfactory interface bonding quality in low temperature and pressure bonding conditions. However, as the coating layer is limited in size to several microns, a large thermal stress generated in the power modules could not be alleviated due to a harsh operating environment and high-temperature reliability changes. This is a substantial issue. As thermal stress significantly affects the lifetime of the power modules, a soft intermediate bonding layer for suppressing the stress applied to the chip die is necessary.

This paper introduced an approach of die-attach bonding technology based on a low-cost and soft intermediate bonding layer of Al sheet in SiC power modules. To achieve interface bonding at low temperature and low pressure, the bonding surfaces of SiC power chip, Al sheet and DBA substrate were sputter-coated by a titanium (Ti) and silver (Ag) metallization layers. The solid Ag-Ag interface was diffusion bonded by a novel mechanism by hillocks generation due to stress self-generation and self-release. In addition, the SiC/Al sheet/ DBA substrate joints were also age-tested at 250 °C for 500 h to evaluate the high-temperature reliability of the joint structures. The shear strength of the joint structure at different temperature bonding was evaluated and hillocks generation and the interface bonding mechanism by surface stress self-generation and self-release were systematically analyzed by scanning electron microscopy (SEM), X-ray diffraction (XRD), and transmission electron microscopy (TEM).

## Results

### Bonding performance of Al sheet joint

**Figure 1:**
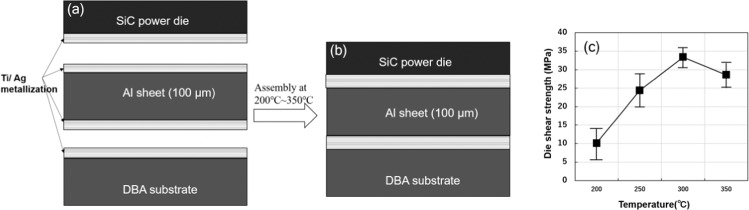
The schematic diagram of the SiC joint structure (**a**) before and (**b**) after assembly, (**c**) the die shear strength of Al sheet joint structure at different temperatures.

Figure 1(a),(b) show the schematic diagram of the SiC joint structure by Al sheet before and after assembly, respectively. Figure 1(c) shows the correlation between the die shear strength of the joint structure and the bonding temperature. At bonding temperatures of 200 °C, 250 °C, 300 °C and 350 °C, the average die shear strength was 10.1 MPa, 24.4 MPa, 33.4 MPa, and 28.6 MPa, respectively. The die shear strength increases with higher temperatures from 200–300 °C and slightly decreases at 350 °C. It is approximately 33.4 MPa at a heating temperature of 300 °C, which is larger than that of traditional Sn-Pb solders (19–24 MPa)^30^ and comparable with Ag sinter joining technology^31,32^. As the Al sheet has excellent thermal conductivity (237 W/(m·K)), electric resistivity (28.2 nΩ·m), and a high melting temperature (660 °C), it can serve as an excellent solution for wide band gap (WBG) semiconductor die-attach modules in high-temperature applications. Selected properties of the Al sheet compared with the Ag thin film bonding, other solders and Ag sinter paste die-attach materials are listed in Table 1. The Ag thin film bonding technology and Ag sinter paste joining technology have some advantages, such as a high melting point and super thermal conductivity. However, their complex printing processes and high costs are major obstacles for industrial applications. The high purity Al sheet bonding technology introduced in this study is more cost-efficient and has a simpler bonding process compared with the Ag thin film bonding technology and Ag sinter paste joining. In addition, the melting point is higher than almost all solder materials, showing better electrical and thermal properties than the solder materials listed in Table 1.

**Table 1:** Material properties of pure Al sheet die-attach compared with other materials.

Materials	Electrical Resistivity (μΩ・cm)	Thermal Conductivity [W/(M・K)]	Melting Point (°C)	Coefficient of Thermal Expansion (µm/(m·K))	Young’s Modulus (GPa)
Ag thin film^25,26^	1.5	429	961	18.9	83
Ag sinter paste^11,12,14^	2.5–10	200–300	961	20.3	32
Pb-5Sn^45,46^	17.4	23	307	30	22
Cu_6_Sn_5_^47,48^	17.5	34.1	415	16.3	85.5
Sn-Ag-Cu^6,7^	11	50	218	21	31
Al sheet (this study)	2.8	237	660	23.1	70

**Figure 2:**
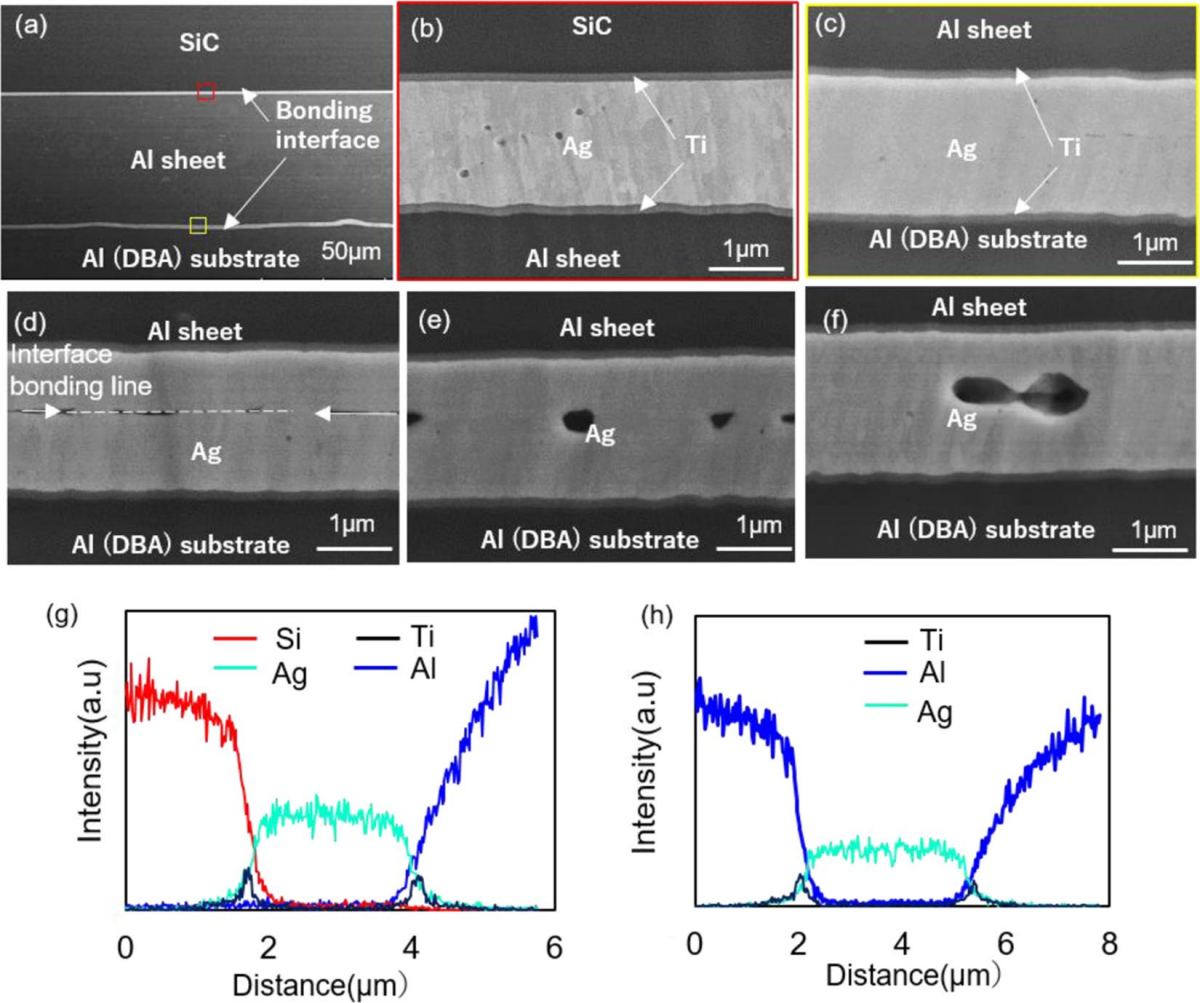
(**a**) Scanning electron microscopy (SEM) image of cross-section of SiC die-attach structure by the high purity Al sheet; (**b**) bonding interface between SiC die and Al sheet, (**c**) bonding interface between Al sheet and directly bonded aluminum (DBA) substrate at 300 °C, and cross-section of the bonding interface between Al sheet and DBA substrate at (**d**) 200 °C, (**e**) 250 °C and (**f**) 350 °C; (**g**) energy-dispersive X-ray diffraction spectroscopy (EDS) line mapping of the interface between SiC die and Al sheet; and (**h**) EDS line mapping of the bonding interface between Al sheet and DBA substrate.

To analyze the interface bonding status at different bonding temperatures and the correlation with die shear strength, the cross-section of the SiC die-attach structure by the Al sheet was observed, as shown in Fig. 2(a). Two bonding interfaces exist: that between the SiC die and the Al sheet, and between the Al sheet and the DBA substrate. The cross-section of the two bonding interfaces at 300 °C are shown in Fig. 2(b),(c). The sputtering coated Ag-Ag layer at both interfaces bonded together without any large gaps. Distinguishing the Ag-Ag bonding interface is very difficult where sputtering coated Ag-Ag changes to an integral thin Ag layer. X-ray system (XVA-160N, Uni-Hite System Corporation, Japan) analysis indicates that large voids are not generated, and the interface bonding ratio is approximately 90%. Figure 2(d)–(f) show the cross-section of the bonding interface between the Al sheet and the DBA substrate at temperatures of 200 °C, 250 °C, and 350 °C, respectively. At a low temperature of 200 °C, numerous gaps appeared at the bonding interface, indicating that the interface did not bond well, which corresponds to a low die shear strength (Fig. 1). Some large voids formed at the Ag-Ag bonding interface at a temperature of 350 °C. Since void formation can quickly lead to crack extensions during the die shear test, die shear strength slightly decreased and was lower than that at 300 °C (Fig. 1). EDS line mapping of the interface between the SiC die and the Al sheet and the bonding interface between the Al sheet and the DBA substrate are shown in Fig. 2(g),(h), respectively. Diffusion among the different layers is indistinct with no intermetallic compound (IMC) layer generation. The Ti layer in this study have some functions which can prevent possible diffusion between the Ag coating layer and the Al sheet. In addition, owing to the coefficient of thermal expansion (CTE) mismatch among Al, Ti, and Ag layers, the Ti layer should improve the surface stress and surface hillocks generation, and thus increase the interface joint strength.

**Figure 3:**
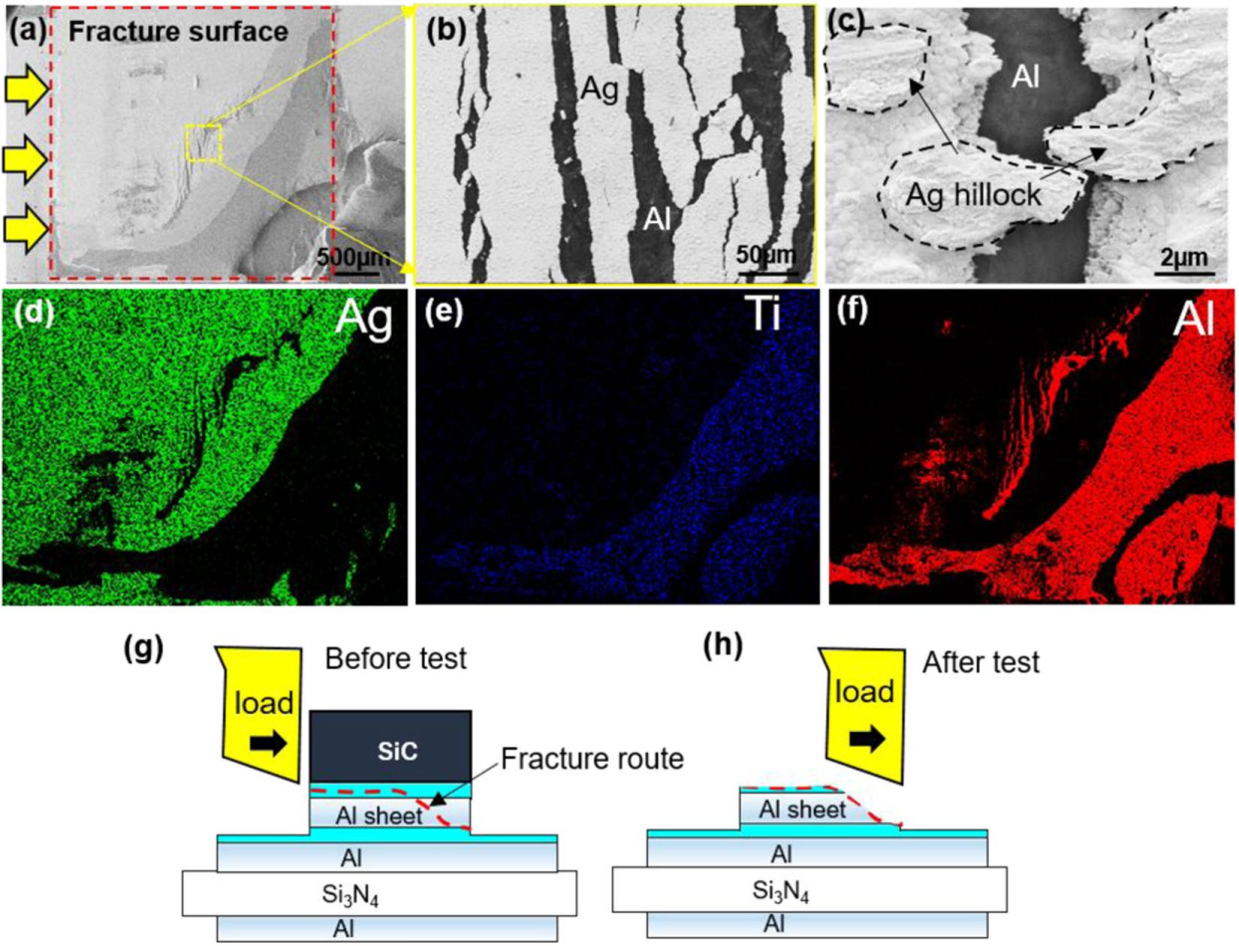
(**a**) Scanning electron microscopy (SEM) image of fracture surface after shear test; (**b**) magnified view of the fracture surface; (**c**) hillock deformation and ductile fracture after shear test. Parts (**d**), (**e**), and (**f**) exhibit energy-dispersive X-ray diffraction spectroscopy (EDS) element mapping of Al, Ag and Ti, respectively; and (**g**) and (**h**) show a schematic of the fracture route before and after the shear test, respectively.

Figure 3 shows the fracture surface after the shear test for the Al sheet joint structure at 300 °C. The direction of the arrow represents the direction of the shear load, as shown in Fig. 3(a). Fractures occurred along the Ag-Ag bonding line at the SiC chip side and with the Al sheet at the final part before completion of the shear test. Figure 3(b) shows a magnified view of the fracture surface where the Ag layer was torn into many pieces by the large deformation of the Al sheet layer during the shear test. Many hillocks exist on the Ag surface. A large deformation occurred at the hillocks in the same direction as the shear load, as shown in Fig. 3(c), meaning that the hillocks were subjected to significant stress and a ductile failure before maximum loading. Figure 3(d)–(f) exhibit EDS element mapping of Al, Ag and Ti, respectively, and confirm the fracture mode. The Ag layer could not be detected with the separate destruction of the Al sheet, which separated from the Ti layer on the Al sheet. This means that the Ag-Ag interface has robust bonding. Figure 3(g),(h) provide a schematic of the fracture route before and after the shear test, respectively.

### Interface bonding mechanism

**Figure 4:**
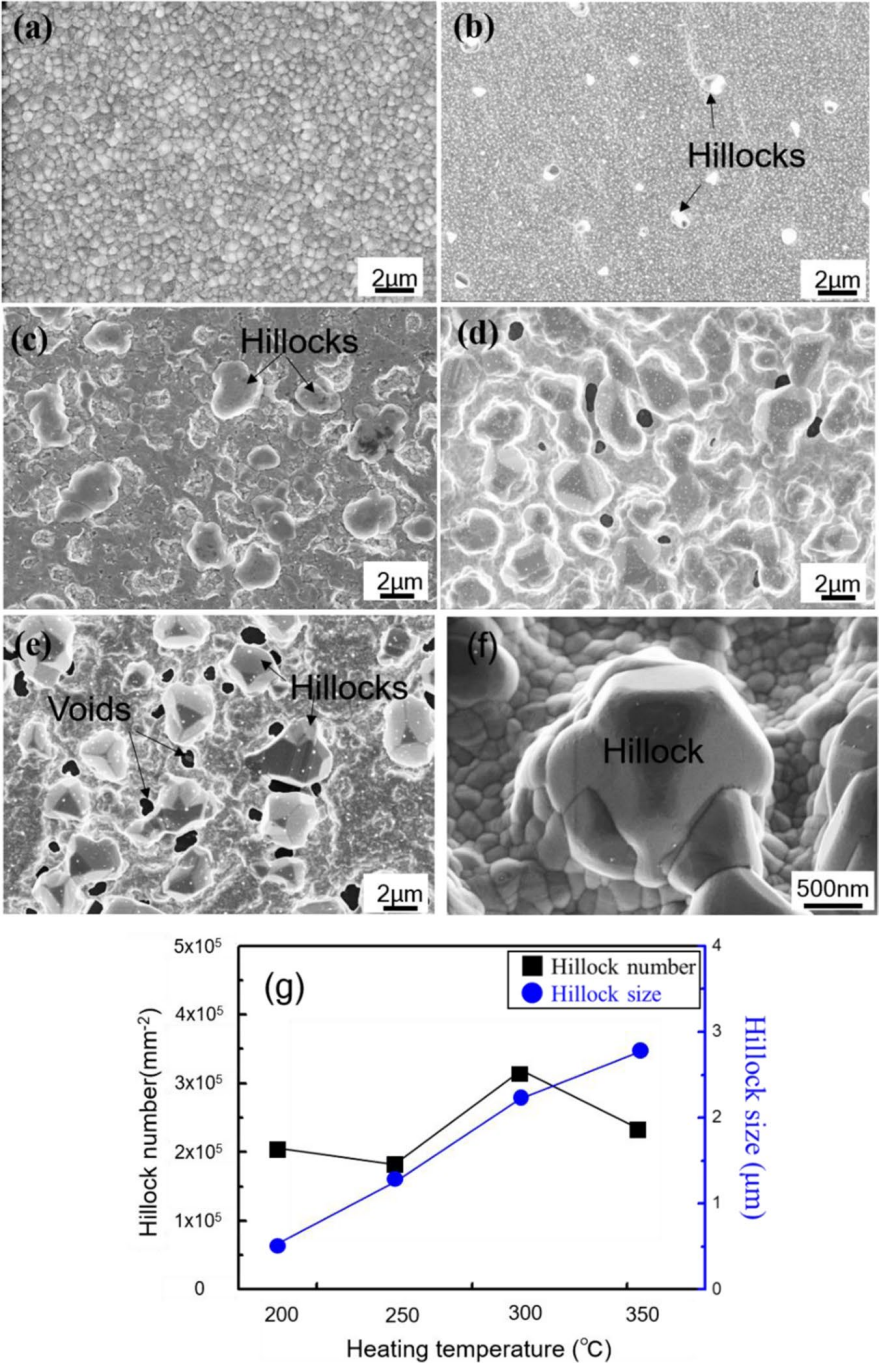
Growth of hillocks on the Ag sputtering-coated Al sheet, (**a**) the initial state of sputtering Ag layer, (**b**) at temperatures of 200 °C, (**c**) 250 °C, (**d**) 300 °C and (**e**) 350 °C, (**f**) the magnified view of the hillock, (**g**) the relationship between number and size of hillocks and temperature.

To understand the die-attach bonding mechanism of the Al sheet, the surface of the Al sheet coated by Ti/Ag was investigated by SEM after heating at temperatures ranging from 200–350 °C for 1 h in air. Figure 4 shows an SEM image of the Al sheet surface coated by Ti/Ag before and after heating. Figure 4(a) shows the top surface of the Ag layer, which has a smooth surface and fine grains. After heating at 200 °C, small hillocks are observed on the Ag layer (Fig. 4(b)). The hillocks become larger and denser with increasing temperature (Fig. 4(c)). Many micron-sized Ag hillocks were generated on the surface of the Al sheet after heating at 300 °C (Fig. 4(d)). Hillock density slightly decreases at 350 °C, and some voids formed on the Ag surface (Fig. 4(e)). Figure 4(f) shows a single Ag hillock of micron-sized diameter and height. The voids may be induced by lateral grain growth of Ag, which consumes a substantial amount of Ag near the Ag hillocks^22^. Void formation should influence interface bonding, causing a decrease in shear strength at 350 °C, as shown in Fig. 1. Correlation between number and size of hillocks and temperature is shown in Fig. 4(g). Growth of these hillocks can be controlled by adjusting the temperature, and thus, higher interface bonding quality may be achieved by optimizing the temperature used. The hillocks play an important role in interface bonding, bridging the interface during heating.

**Figure 5:**
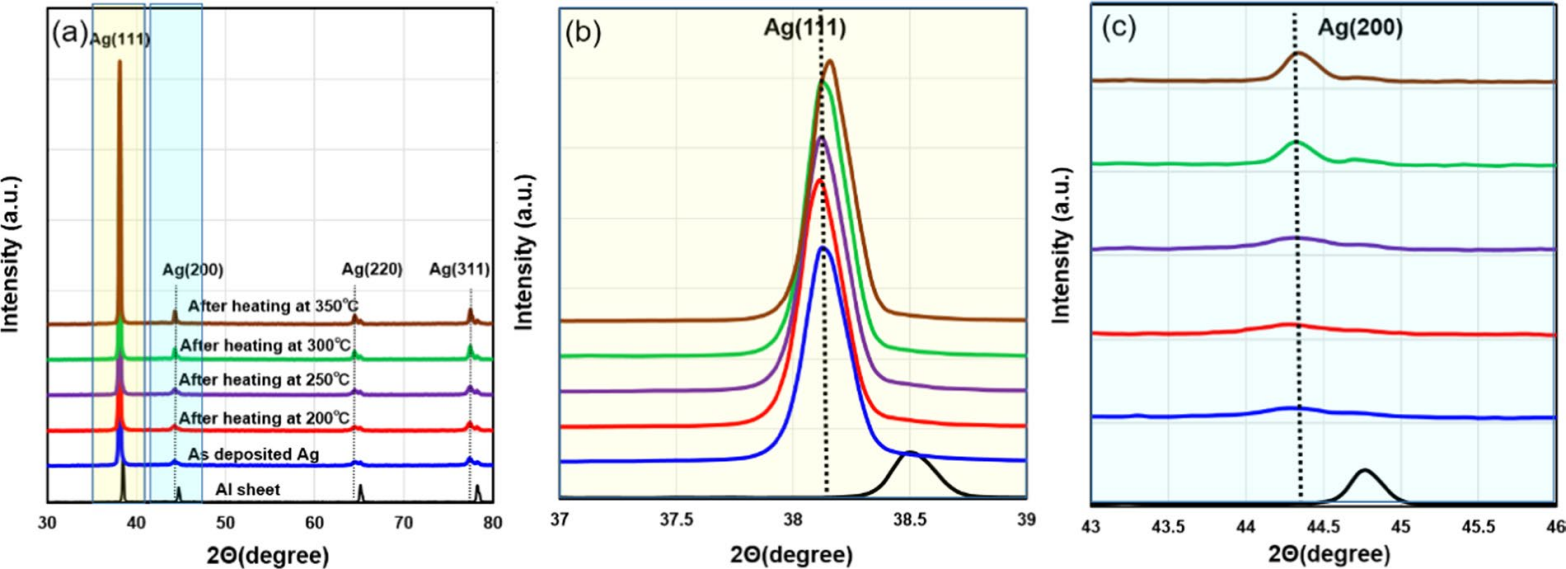
(**a**) X-ray diffraction (XRD) patterns of the top surface of the Ag layer with different heating processes; (**b**) incident angle plotted from 37° to 39°; and (**c**) incident angle plotted from 43° to 46°.

The heat treatment effect of the Ag-coated Al sheet on the growth of hillocks was analyzed by XRD. Figure 5(a) shows the XRD patterns of the top surface of the Ag layer. The substrate was heated from 200 °C to 350 °C and compared with the case without heating treatment. Ag (111) has the highest diffraction peak in XRD analysis. With an increase in the preheating temperature, the peak angle of Ag tends to increase. A change in Ag (111) is observed in Fig. 5(b), where the incident angle is plotted from 37° to 39°. This indicates that heating may change the grain structure of the Ag coating layer. The Ag peak (200) becomes sharp with a higher heating temperature, as shown in Fig. 5(c), which means that the grain boundary crystallinity of Ag improves during heating. Based on the XRD results, IMC generation did not occur during heating at any of the temperatures tested.

Generation of hillocks is attributed to stress migration due to CTE mismatch of each layer; residual thermal stress occurs in the compressive direction, causing the diffusion of atoms from higher to lower compressive stress regions^33,34^. Growth of hillocks has also been reported in the mechanism of nano-volcanic eruption, where Ag-O fluids at grain boundaries are squeezed out toward the free surface with the release of compressive stress and Ag nanoparticle generation^35,36^. In this study, surface stress self-generation and self-release mechanisms on the Al sheet were analyzed by investigating the surface stress of the as-deposited Ti/Ag layer and during the heating process. The residual stresses in the Ag layer of the as-deposited Ti/Ag layer on the Al sheet were measured by XRD. A tensile stress of 166 MPa on the Ag top surface was calculated. Due to compressive stress caused by CTE mismatch among the Ag, Ti and Al layers, which possess different material properties including CTE, thermal stress during heating can be calculated by the following equations^37^:

1 $${\sigma }_{x}={\sigma }_{y}\approx \frac{{E}_{Ag}\Delta T({\alpha }_{1}-{\alpha }_{2})}{(1-{v}_{Ag})}$$

2 $${\sigma }_{z}\approx 0$$

3 $${\sigma }_{Ag}=\frac{{\sigma }_{x}+{\sigma }_{y}+{\sigma }_{z}}{3}$$

where ${\sigma }_{x}$ and ${\sigma }_{y}$ are the thermal stresses in the horizontal direction and ${\sigma }_{z}$ is the thermal stress in the vertical direction. E_Ag_ (76 GPa) is Young’s modulus of the Ag layer, and V_Ag_ is Poisson’s ratio of the Ag layer. ∆T is the temperature difference from room temperature to heating temperature. α_1_ (= 8.9 µm/(m•k)) and α_2_ (=19.6 µm/(m•k)) are the CTE of Ti and of Ag, respectively. The released residual stress was calculated as −151.3 MPa, −187.6 MPa, −229.36 MPa and −293.6 MPa when the heating temperature was 200 °C, 250 °C, 300 °C and 350 °C, respectively. The higher heating temperature corresponds to greater stress release, which causes the generation of Ag hillocks, as shown in Fig. 4, and bonds the interfaces of the Al sheet joint structure.

**Figure 6:**
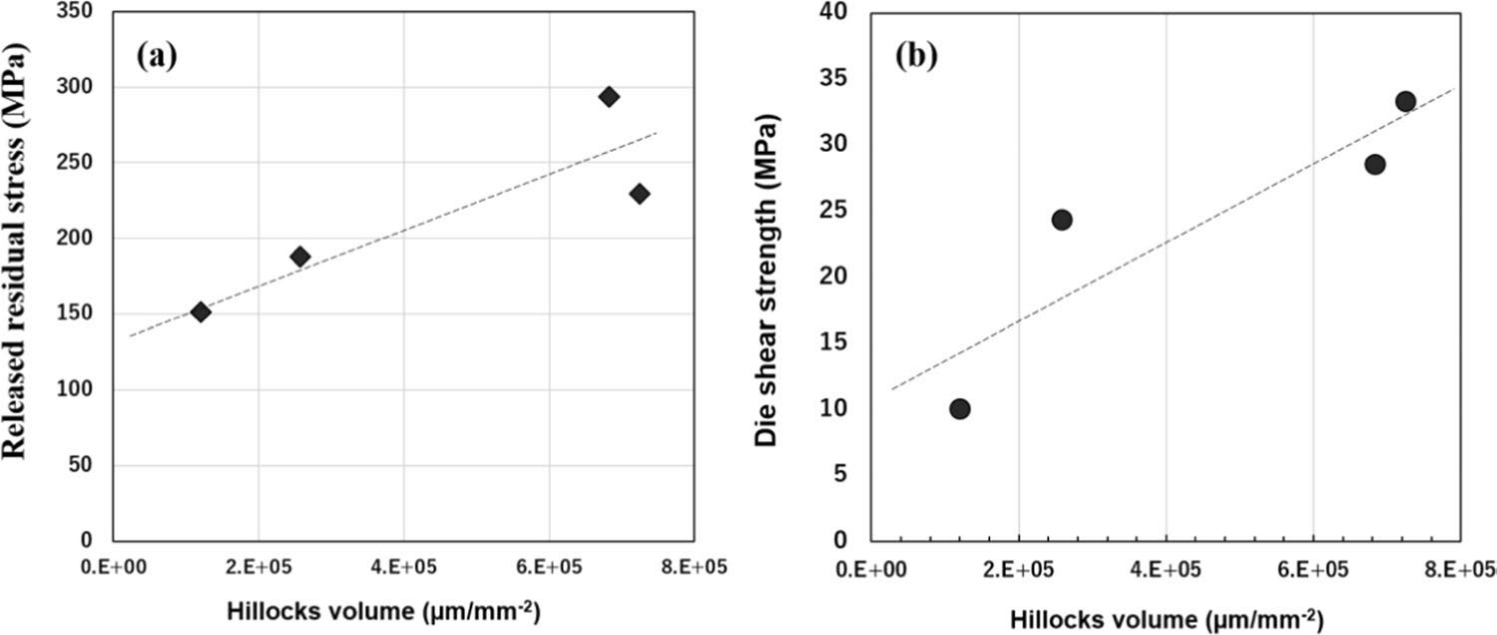
(**a**) the relationship between the released residual stress and the hillocks volume, (**b**) the relationship between the die shear strength and the hillocks volume.

Figure 6(a) shows the relationship between the released residual stress and the hillocks volume (hillocks number *hillocks average size), where the released residual stress has a positive proportional relationship with the hillocks. Figure 6(b) shows the relationship between the die shear strength and the hillocks volume, which also shows a positive relationship. Thus, the die shear strength was influenced by the hillocks generation which largely depends on the surface stress release.

**Figure 7:**
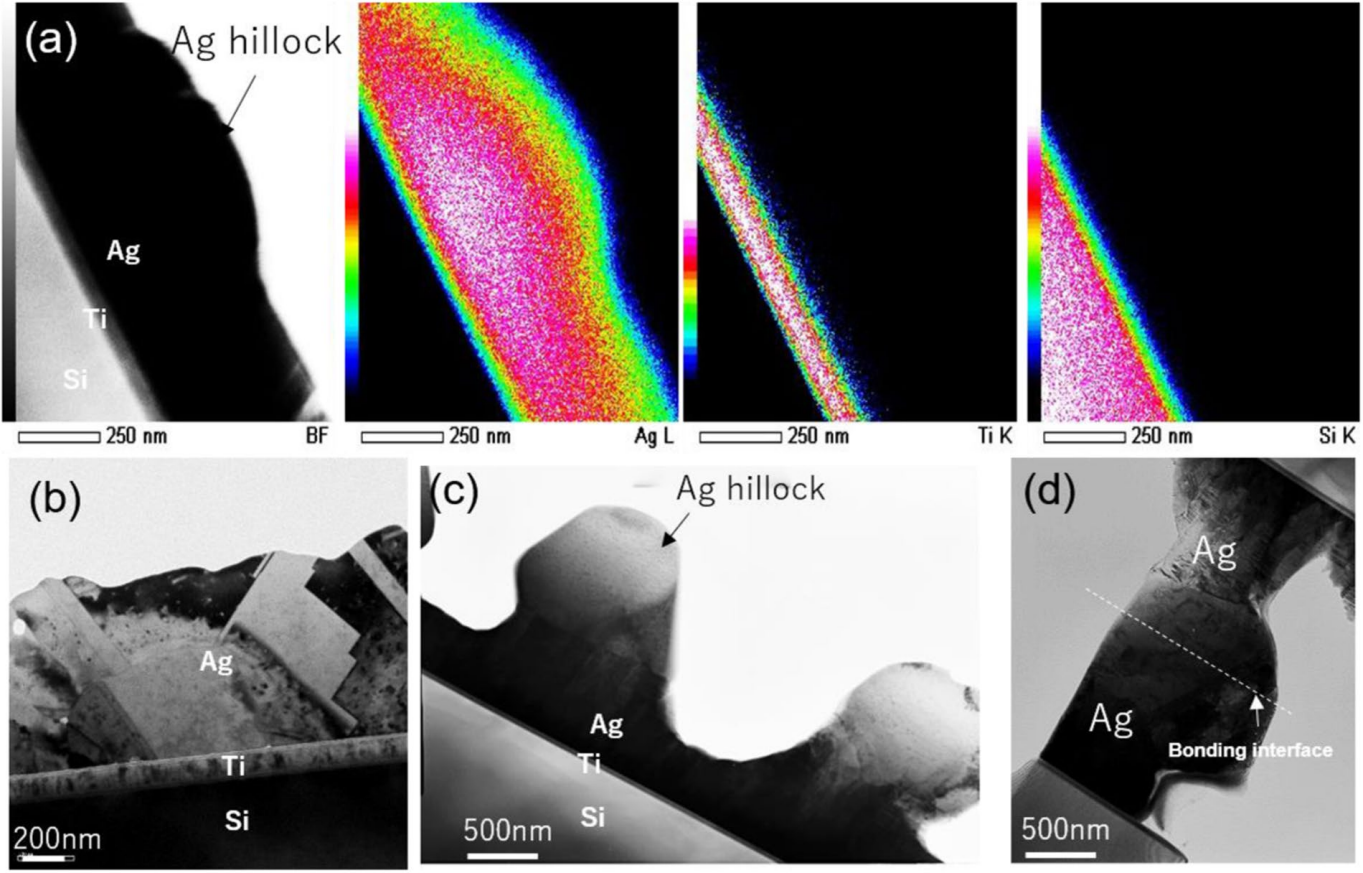
(**a**) Transmission electron microscopy (TEM) image of Ag hillock growth and energy-dispersive X-ray diffraction spectroscopy (EDS) mapping after heating at 250 °C for 5 min; (**b**) TEM image of the sputtered Ti and Ag layer on Si substrate in initial state; (**c**) hillock growth after heating; and (**d**) Si bonding structure by sputtering Ag-Ag layer after heating at 250 °C for 1 h.

To further understand hillock growth and Ag-Ag interface bonding, these phenomena were observed by TEM (JEM-ARM200F, JEOL) at 200 KV. Ti/Ag was sputtered on the Si chips and Si substrate to easily fabricate a TEM sample. Figure 7(a) shows the TEM cross-section of the Ti/Ag layers coated on the Si substrate after heating for 5 min at 250 °C. The hillock has grown after 5 min, as shown in the EDS mapping image. In addition, the Si chip and substrate were simultaneously mounted and heated at 250 °C for 1 h with the same pressure as in the Al sheet bonding. Figure 7(b) exhibits the TEM image of the sputtered Ti/Ag layer on the Si substrate in the initial state. Figure 7(c) shows hillock growth after heating. The Ag hillock has a single crystal structure and grows from the grain boundary of the Ag sputtering layer, as reported in previous studies^38,39^. Figure 7(d) shows the Si-Si joint structure by the sputtered Ti/Ag layer. An Ag-Ag layer was formed to an integrally Ag thin film without any gaps at the bonding interface. Therefore, the novel bonding mechanism using Al surface stress generation and release with surface hillock generation can provide a new solution for SiC die-attach devices in high-temperature applications to achieve interface bonding at low temperature and pressure. As Ag hillocks are generated on the entire Al surface with a relatively even distribution, the bonding technology can cover a large area for a large die size.

### High temperature reliability

**Figure 8:**
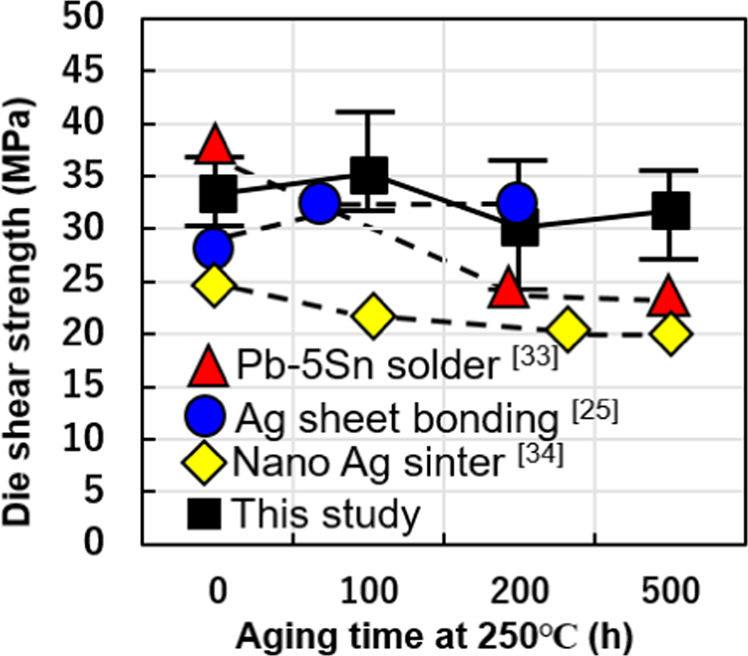
High-temperature reliability of Al sheet bonding structure maintained at 250 °C for different lengths of time and a comparison with other bonding technologies.

Figure 8 shows the die shear strength of the Al sheet joint structure maintained at 250 °C for 100 h, 200 h, and 500 h. Die shear strength does not undergo a substantial change with changes in aging time. It slightly decreases from the as-bonded strength of 33.4 MPa to 31.2 MPa after 500 h, which suggests that the bonding technology has excellent high-temperature reliability. The high-temperature stability is comparable to that of Pb-5Sn solder^40^, joined sintered Ag nanoparticles^41,42^ and Ag foil bonding technology^25^, as shown in Fig. 8.

**Figure 9:**
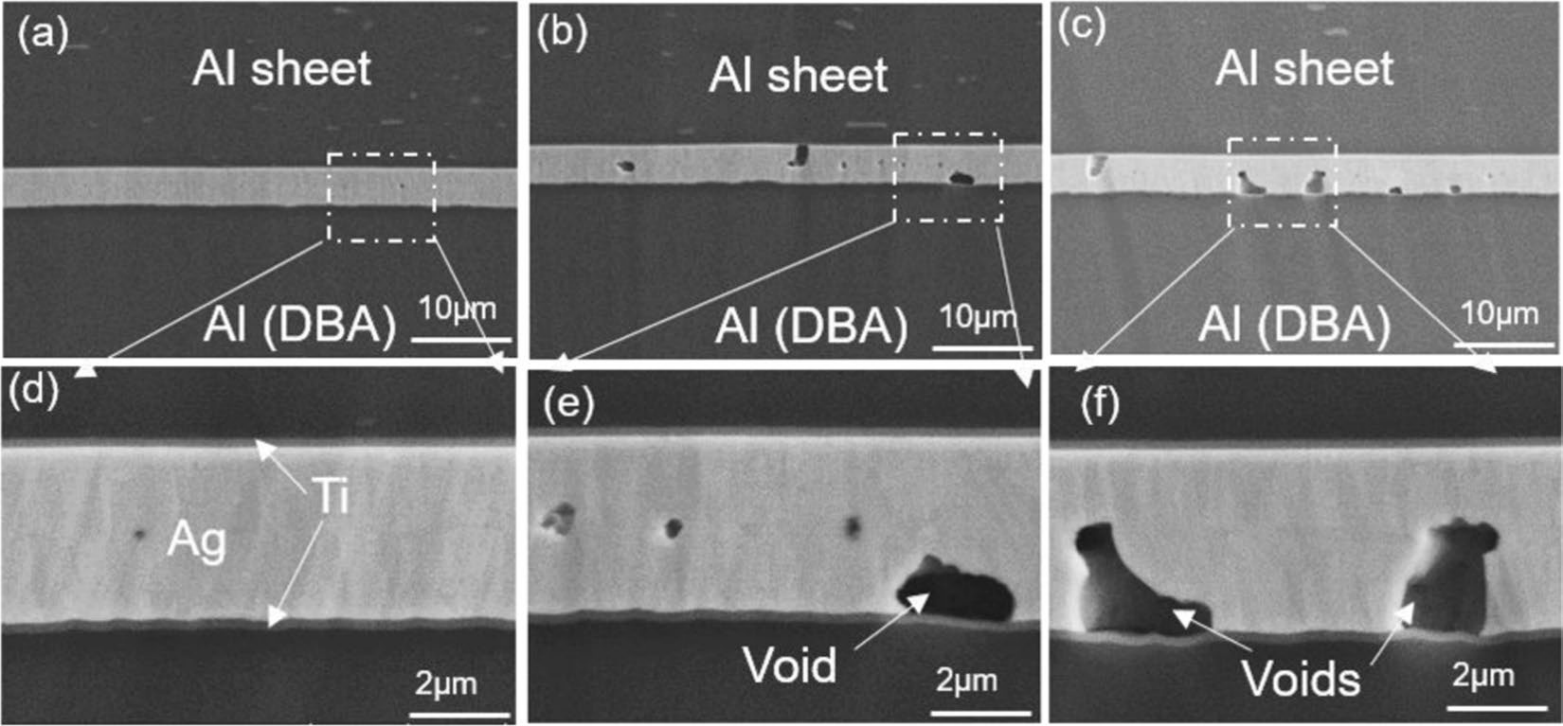
Scanning electron microscopy (SEM) image of cross-section of the bonding structure between Ag sheet and directly bonded aluminum (DBA) substrate after aging for (**a**)100 h, (**b**) 200 h, and (**c**) 500 h; (**d**), (**e**) and (**f**) show a magnified view of the bonding interface that corresponds to (**a**), (**b**) and (**c**), respectively.

Figure 9(a)–(c) show the SEM image of the cross-section of the bonding structure between the Ag sheet and the DBA substrate after aging for 100 h, 200 h, and 500 h, respectively. The sputtered Ag-Ag layer remains well bonded where deformation of the Al sheet and DBA substrate is not distinct, as shown in Fig. 9(d). There is no clear diffusion between different layers, and IMC layer generation and the interface do not exhibit any delamination even after aging for 500 h. Voids appeared in the sputtered Ag layer after aging for 200 h (Fig. 9(e)), and more voids appeared with an increase in the aging time (Fig. 9(f)). The appearance of voids in the sputtered Ag layer is a de-wetting phenomenon from Ti, as reported in^43^. The Ag atoms in the Ag metallization layer are passively absorbed into the Ag bonding interface due to stress migration^44^.

**Figure 10:**
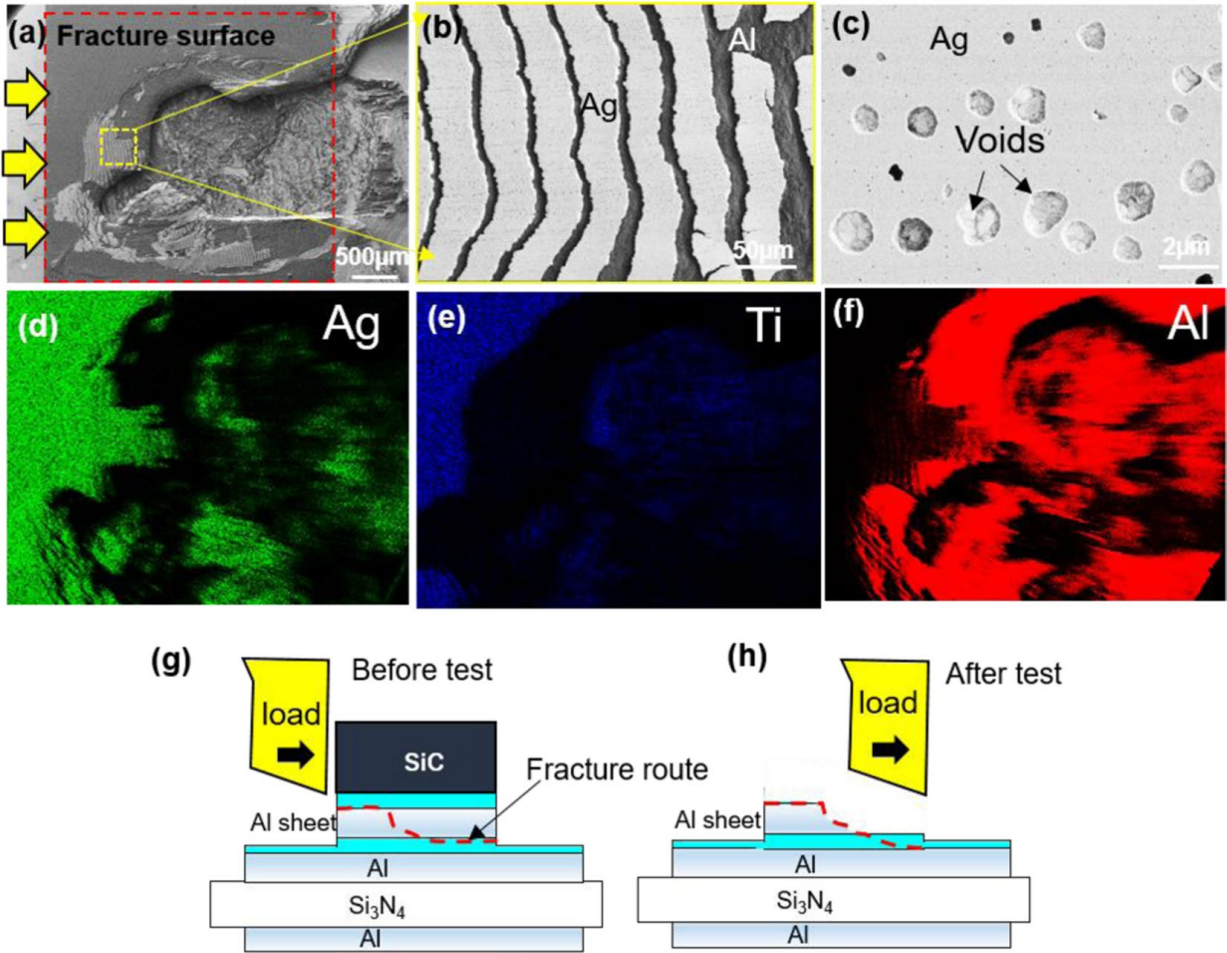
Scanning electron microscopy (SEM) image of fracture surface of the Al sheet joint structure at 250 °C after aging for 500 h; (**b**) magnified view of the fracture surface; and (**c**) voids on the Ag layer and the ductile fracture after shear test. Parts (**d**), (**e**), and (**f**) exhibit energy-dispersive X-ray diffraction spectroscopy (EDS) element mapping of Al, Ag, and Ti, respectively; and (**g**) and (**h**) show a schematic of the fracture route before and after the shear test, respectively.

Figure 10(a) shows the fracture surface of the Ag sheet joint structure after aging at 250 °C for 500 h. The fracture primarily occurred inside the Al sheet with a large deformation, where almost one third of the Al sheet was separated, meaning that the Al sheet was subjected to great stress before the bonded interface fractured. The fracture morphology on the top Ag sheet shows a ductile deformation, tearing the Ag layer on the Al sheet surface into many pieces (Fig. 10(b)). In addition, many voids are generated on the Ag layer, as shown in Fig. 10(c), which corresponds to the de-wetting phenomena from Ti, as shown in Fig. 9(f). Therefore, the Ti and Ag layer may separate during the shear test due to generation of voids, which causes a slight decrease in die shear strength, as shown in Fig. 8. Figure 10(d)–(f) exhibit the EDS element mapping of Al, Ag and Ti, respectively, where the Al element at the separated part of the Al sheet corresponds to the surface of the DBA substrate. Figure 10(g),(h) provide a schematic of the fracture route before and after the shear test, respectively.

## Methods

High purity aluminum (99.99%) with a thickness of 100 µm was applied as a die-attached layer, cut into 4.0 mm × 4.0 mm pieces. A SiC chip (3.0 mm × 3.0 mm, t = 0.45 mm) and DBA substrates were prepared. The backside of the SiC and the top surface of the DBA were orderly and were metalized by a Ti layer with a thickness of 0.2 μm and an Ag layer with a thickness of 1 μm. Both sides of the Al sheets were sputtered by the Ti/Ag layer using the same sputtering process. After sputtering, the Al sheet was sandwiched between the SiC die and the DBA substrate. The sandwiched sample SiC, Al sheet, and DBA were heated for 1 h on a hot plate under 1 MPa pressure to temperatures ranging from 200 °C to 350 °C. After heating, two Ag-Ag bonding interfaces were observed in this joint structure. To evaluate the high-temperature reliability of the joint structure using the Al sheet, the joint samples were age-tested at 250 °C for 500 h.

The bonding quality of the joint samples at each aging time was measured by die-shear tests (XD-7500, DAGE). The height from the DBA substrate surface was set to 100 μm, equal to the thickness of the Al sheet. The shear load was parallel to the SiC chip. The shear rate was 50 μm/s. Five joint samples from each bonding condition were measured to obtain average die shear strengths. The bonding process and mechanism of the die-attach structure of the Al sheet on the DBA structure was explored using scanning electron microscopy (SEM), energy-dispersive X-ray diffraction spectroscopy (EDS), X-ray diffraction (XRD), and transmission electron microscopy (TEM). The cross-section of the sintered Ag joint was prepared by an ion milling polishing machine (IM 4000; Hitachi, Japan), and the microstructure of a sintered Ag joint was observed by SEM (SU-8020; Hitachi, Japan). The atomic distribution at the bonded interface was analyzed by EDS (Hitachi, Japan). The bonding mechanism was analyzed by TEM (JEM-ARM200F, JOEL, Japan) at 200 KV. XRD was implemented with a scintillation detector and rotating Cu Kα radiation (λ = 0.154 nm) to analyze the residual stress after Ti/Ag sputtering and the peak intensities of the Al sheet with sputtering layers at different heating temperatures in the angular range from 20° to 90° (2θ).

## Conclusions

This work presents a novel attractive bonding technology by a pure Al sheet for a low-cost die-attach material with high reliability for SiC power modules in high-temperature applications. An initial die shear strength of 33.4 MPa was achieved in low temperature and pressure bonding conditions, and a die shear strength of 31.2 MPa was maintained after aging at 250 °C for 500 h, which confirmed that the Al sheet joint possesses excellent thermal stability. In contrast with the solid interface diffusion bonding mechanism, the novel die bonding of the Al sheet by stress self-generation and self-release was systematically investigated by SEM, XRD, and TEM. Tensile stress was self-generated at the sputtering stage and released to generate Ag hillocks on the Al sheet during heating. Interfaces of the Al sheet joint structure were bonded by the growth of Ag hillocks with a robust interface strength. Since the Al sheet has low cost and excellent properties, such as thermal conductivity (237 W/(m·K)), electric resistivity (28.2 nΩ·m) and thermal stability, this die-attach bonding technology is an attractive alternative for wide band-gap devices that require high-temperature performance.

## Supplementary information

Supplementary information.

## Data Availability

The raw and processed data required to reproduce these results are available by contacting the authors.
