# Supplementary material for: Low temperature SiC die-attach bonding technology by hillocks generation on Al sheet surface with stress self-generation and self-release
Source: Sci Rep. 2020 Jun 3;10:9042. doi: 10.1038/s41598-020-66069-8 (PMC7270210; doi:10.1038/s41598-020-66069-8)
Supplement: Supplementary file 1 — Supplementary information. [file 41598_2020_66069_MOESM1_ESM.docx]

**Supporting information**

**Low temperature SiC die-attach bonding technology using pure Al sheet by surface stress self-generation and self-release**

Chuantong Chen^a^*,Katsuaki Suganuma^a^

^a^ Institute of Scientific and Industrial Research, Osaka University, Mihogaoka 8-1, Ibaraki, Osaka 567-0047, Japan

*E-mail: [chenchuantong@sanken.osaka-u.ac.jp](mailto:chenchuantong@sanken.osaka-u.ac.jp)


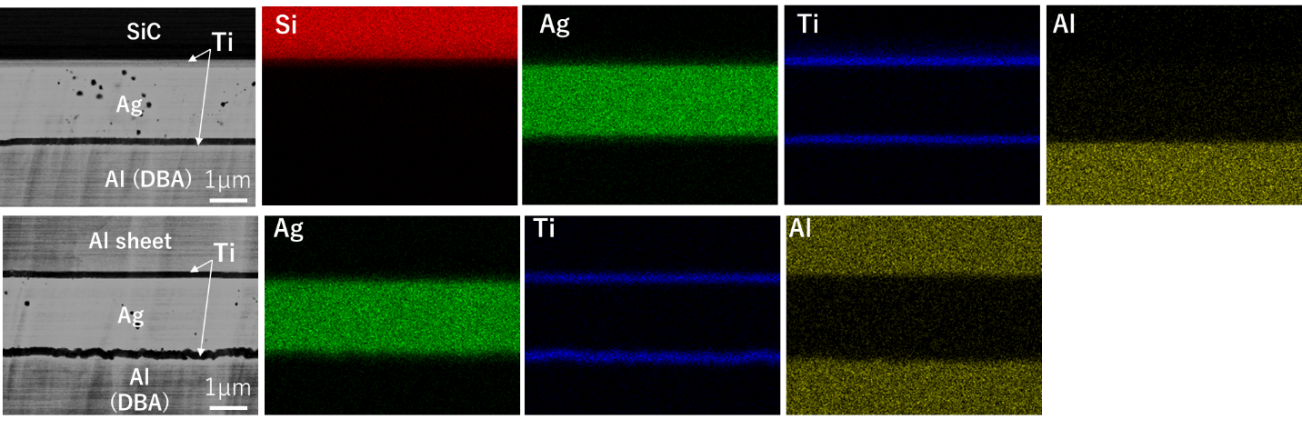


Figure S1. The bonding interface between SiC die and Al sheet and the bonding interface between Al sheet and DBA substrate and its EDS element mapping. Al sheet and DBA substructure was slight deformation but not clear diffusion between different layers and intermetallic compound (IMC) layer generation.
